# Redirecting the immune response towards immunoprotective domains of a DNABII protein resolves experimental otitis media

**DOI:** 10.1038/s41541-019-0137-1

**Published:** 2019-10-14

**Authors:** L. A. Novotny, S. D. Goodman, L. O. Bakaletz

**Affiliations:** 1 Abigail Wexner Research Institute at Nationwide Children’s Hospital, Columbus, OH 43205 USA; 2 The Ohio State University College of Medicine, Columbus, OH 43210 USA

**Keywords:** Vaccines, Bacterial pathogenesis, Vaccines

## Abstract

The chronicity and recurrence of many bacterial diseases is largely attributable to the presence of a biofilm, and eradication of these structures is confounded by an extracellular DNA-rich matrix. DNABII proteins, including integration host factor (IHF), are critical components of the matrix formed by all human pathogens tested to date. Whereas the natural adaptive immune response to IHF is against non-protective epitopes within the carboxyl-terminal region, antibodies against the DNA-binding “tips” induce biofilm collapse. We designed a “tip-chimer” immunogen to mimic the DNA-binding regions within the α-subunit and β-subunit of IHF from nontypeable *Haemophilus influenzae* (IHF_NTHi_). Re-direction of the natural adaptive immune response toward immunoprotective domains disrupted NTHi biofilms in vitro and in an experimental model of otitis media. Our data support the rational design of a powerful therapeutic approach, and also that of a DNABII-directed vaccine antigen that would avoid augmentation of any pre-existing natural, but nonprotective, immune response.

## Haemophilus influenzae: Targeting the tip for biofilm collapse

Bacterial biofilms are characterized by the presence of a protective extracellular polymeric substance (EPS) that incorporates both eDNA and members of the DNABII family of bacterial DNA-binding proteins. Antibodies against the “tips” of these DNA binding-domains can cause biofilm collapse, but these epitopes are masked from the host adaptive immune system when bound to eDNA, making biofilm eradication difficult. Here, the team led by Lauren Bakaletz used a chimeric peptide to generate tip-specific antibodies against nontypeable *Haemophilus influenzae* to treat biofilms in vitro and in vivo. The “tip-chimer” contained the immunoprotective domains from the DNA-binding tips of a DNABII protein, integration host factor (IHF), expressed by nontypeable *Haemophilus influenzae*. The consequent antibodies disrupted *H. influenzae* biofilms in vitro and were used to treat a chinchilla model of experimental otitis media when inoculated directly into the middle ear, resulting in reduced bacterial load and clearance of already established mucosal biofilms. These findings suggest that redirecting the host adaptive immune response towards the immunoprotective tips of DNABII proteins could provide a strategy to eradicate biofilms caused by various pathogens that produce these proteins.

## Introduction

Biofilms are implicated in 65–80% of all chronic bacterial diseases, the spectrum of which include persistent infections in soft tissues, on mucosal surfaces, and on indwelling medical devices.^1^ Collectively, there is a tremendous economic burden and an immeasurable negative impact on patient quality of life associated with these chronic and recurrent biofilm infections.^2^ The recalcitrant nature of these multicellular, and often multispecies, bacterial communities to antibiotic therapy or clearance by the host immune system is due, in part, to the presence of a self-produced extracellular polymeric substance (EPS) that enshrouds the resident bacteria and is comprised protein, polysaccharide, and nucleic acid.^3,4^ Development of therapeutic strategies to eradicate these formidable structures that focus on components within the EPS are complicated by the varied and complex composition of this matrix among biofilms formed by different bacterial species.^5^ Identification of a common constituent within the EPS could have profound value in the rational design of biofilm-directed preventative and therapeutic approaches.

An abundant component of the EPS produced by a diverse array of bacterial species is extracellular DNA (eDNA), released by bacteria upon cell lysis or via active mechanisms.^4,6–11^ This eDNA is uniquely organized into a lattice-like structure, which provides critical structural support to the biofilm.^6^ An additional and essential component of the biofilm structure is the DNABII family of bacterial DNA-binding proteins, which include just two members: integration host factor (IHF) and histone-like protein (HU)^12^ which are ubiquitous among all eubacteria.^13^ Classically studied for their intracellular roles as a DNA architectural element in multiple types of nucleoprotein interactions, DNABII proteins also play a critical role extracellularly in the formation and maturation of bacterial biofilms.^14,15^ Via virtue of being situated at each crossed strand of eDNA within the EPS, this localization of DNABII proteins imparts structural stability to the biofilm.^14,16,17^ Exposure of bacterial biofilms to antibody against DNABII proteins destabilizes the eDNA matrix and leads to collapse of the biofilm structure.^14,18^ The mechanism for this outcome is induction of an equilibrium imbalance as free DNABII proteins are first sequestered, by formation of an antibody complex via their DNA-binding domain. This series of events prevents eDNA rebinding and further leads to a shift of these linchpin proteins away from the biofilm matrix with subsequent structural collapse.^16^ Direct contact of antibody with the biofilm is not required for this collapse, and importantly, the newly released bacteria are significantly more susceptible to the action of previously ineffective antibiotics.^16^ Notably, antibodies against IHF induce: (1) collapse of biofilms formed by all 21 diverse pathogens tested to date in vitro including each of the high priority ESKAPE pathogens;^17,19–21^ (2) prevents the formation of and disrupts biofilms pre-formed by nontypeable *Haemophilus influenzae* (NTHi) biofilms on surgical resorbable material in vitro;^22^ (3) dissolves sputum solids collected from patients with cystic fibrosis^23^ and exudate specimens recovered from the auditory canal of children with post-tympanostomy tube otorrhea;^24^ (4) detects the presence of DNABII proteins within middle ear fluids retrieved from children with otitis media with effusion^25^ and at the site of incision after cesarean section delivery in obese women at risk for skin infection;^26^ (5) disrupts biofilms and thereby induces rapid disease resolution in an experimental model of NTHi-induced otitis media,^14,16^ as well as an in osteolytic model of *Aggregatibacter actinomycetemcomitans*-induced peri-implantitis;^21^ and (6) prevents the development of experimental otitis media in a polymicrobial model of ascending disease.^27^ Thus, due to the ubiquitous nature of bacterial DNABII proteins, and the significant ability of antibodies directed against these proteins to disrupt biofilms formed by all bacterial pathogens tested to date, a DNABII protein-directed therapeutic regimen or immunization strategy has potential for broad efficacy and utility.

However, one question that confounded us was why the adaptive immune system did not generate antibodies against DNABII proteins present within pathogenic biofilms and thereby eradicate these structures naturally? We hypothesized that this might be due to the fact that when IHF or HU was bound to eDNA within a biofilm matrix, the immunoprotective domains of these proteins were perhaps masked by their physical interaction with the eDNA. In a series of in vitro and in vivo studies, we showed that this hypothesis was indeed correct.^16,18^ The DNABII family function as dimers of identical or homologous subunits and have a strong preference to bind to, then bend DNA, but also have a particularly high affinity for pre-bent DNA such as Holliday junctions.^12^ Initiation of DNA bending occurs through intercalation of a proline within the antiparallel β-ribbon “tips” of each subunit.^12^ These interactions sterically occlude the DNA-binding tip regions within IHF and HU, whereas the region referred to here as the “tail”, that includes the second of two alpha helices, are more exposed to the immune system.

**Fig. 1:**
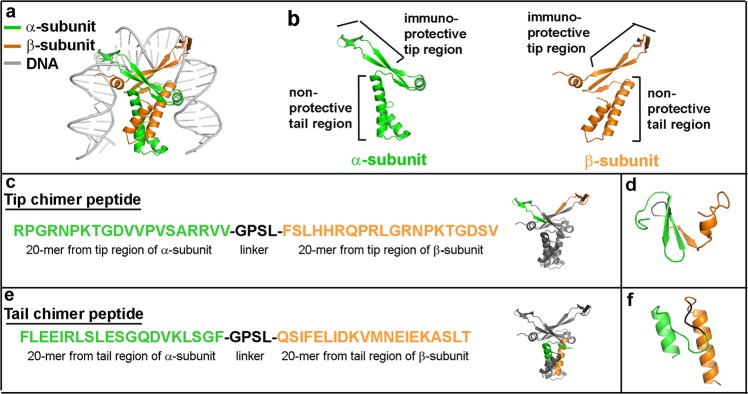
Strategy used in earlier work to design chimeric peptide immunogens against domains we defined as either immunoprotective (e.g., within the DNA-binding tips) or as nonprotective (e.g., within the tail regions) of IHF. **a** PyMOL-rendered model to distinguish between α-subunit and β-subunit that comprise IHF_NTHi_ reveal occlusion of the DNA-binding tip region within each subunit upon binding to DNA. **b** Localization of immunoprotective domains and non-protective domains within the α-subunit and β-subunit of native IHF_NTHi_. **c** Amino acid sequence of a chimeric peptide immunogen that targets immunoprotective domains within the α-subunit and β-subunit of IHF_NTHi_, called “tip chimer”. PyMOL-rendered model depicts location of origin for each peptide domain as mapped on to the deduced structure of native IHF_NTHi_. **d** New model for the tip chimer peptide which predicted retention of structural characteristics ascribed to the DNA-binding tip domain within native IHF protein. Domain from α-subunit is green, domain from β-subunit is orange, and GPSL linker peptide is black. **e** Amino acid sequence of a chimeric peptide immunogen that targets non-protective domains within the α-subunit and β-subunit of IHF_NTHi_, called “tail chimer” and location of origin on native IHF_NTHi_. **f** New predictive structural model for tail chimer peptide. Domain from α-subunit is green, domain from β-subunit is orange, and GPSL linker peptide is black

Antibodies induced by immunization with IHF that is pre-bound to DNA (the form found within biofilms) do not disrupt bacterial biofilms in vitro or in vivo, however, antibodies generated by immunization with purified native IHF do indeed induce rapid and substantial biofilm collapse.^14,16,17,20^ Prior epitope-mapping work revealed a potential mechanism for these observations in that when we immunized chinchillas with IHF that was pre-bound to eDNA, i.e., as present within in a biofilm (Fig. 1a), this resulted in antibodies directed against the long alpha helices within the tail regions of the α-subunit and β-subunit (Fig. 1b).^16^ In contrast, immunization with native IHF-induced antibodies primarily against the DNA-binding antiparallel β-ribbons within the tip regions of the α-subunit and β-subunit of the protein (Fig. 1b). Functionally, antibody directed against the DNA-binding tip regions within either the α-subunits or β-subunits of IHF disrupt bacterial biofilms in vitro and in vivo; however, those against the tail region of IHF are ineffective.^16,18^ Moreover, we reported earlier that a cocktail of antibodies against the DNA-binding tip regions of both subunits (e.g., anti-α-subunit tip + anti-β-subunit tip) showed an additive biofilm disruption effect over that achieved with antibodies against either subunit when used individually.^18^ Thus, based on these data collectively, we proposed that the natural adaptive immune response to DNABII proteins (when they are present within a pathogenic biofilm) is predominantly directed against the more accessible and immunodominant, but non-protective, epitopes within the tail region and thereby, bacterial biofilms persist in the host despite immune recognition of the DNABII proteins.

To develop a protective immunogen, we reasoned that we would need to re-direct the immune response specifically toward immunoprotective domains within the tip regions of IHF, as use of whole native protein was most likely to augment any pre-existing, non-protective response already directed toward the tail regions due to the principles of “original antigenic sin”.^28^ As such, in a recent publication, we reported the design and initial testing of a chimeric peptide that incorporated 20-mer immunoprotective domains from within the DNA-binding tips of both the α-subunits and β-subunits of IHF, joined by a 4-mer linker peptide, the sequence of which permits flexibility between the incorporated epitopes.^29^ This strategy was undertaken to not only address the location of the immunoprotective tip domains but also to take advantage of our already-existing knowledge that whereas antisera directed against either the α-tip or the β-tip alone were highly effective both in vitro and in vivo, the use of a cocktail of these antisera had been found to be even more so, as we have already published.^18^ This “tip chimer” peptide was designed based on native IHF expressed by nontypeable *Haemophilus influenzae*, which is again used herein as a model bacterium due to its prevalence as a human pathogen and capacity to form recalcitrant biofilms in the course of diverse chronic and recurrent upper and lower respiratory tract diseases.^30,31^

This work attempts to further advance our development of this chimeric immunogen to now describe the ability of antibodies against the tip chimer peptide to disrupt biofilms both in vitro and in an experimental model of otitis media when tested in direct comparison with either antibodies to purified native IHF_NTHi_ or to antibodies directed against a similarly designed “tail chimer peptide” (used here as a negative control).

## Results

### Strategy used in our original design of a chimeric synthetic peptide immunogen

To reiterate the above, we provide a schematic of how we designed a chimeric synthetic peptide antigen to focus the adaptive immune response exclusively toward the immunoprotective domains within the DNA-binding tip regions of both α-subunit and β-subunit of IHF, and away from the non-protective domains within the tail region (Fig. 1b). This chimeric synthetic peptide immunogen (called “tip chimer”) incorporated amino acids that represented the established immunoprotective 20-mer segments of the α-subunit and β-subunit of IHF_NTHi_ (Fig. 1c). These 20-mer segments were joined via GPSL linker peptide to induce a turn and therefore permit flexibility between the incorporated epitopes.^29^ As a negative control, a “tail chimer” that mimicked immunodominant, but non-protective epitopes within the long alpha helices in the “tail” region of the α-subunit and β-subunit of IHF_NTHi_ are depicted here (Fig. 1e).

To expand on these original designs, here we have added computer algorithmic analyses^32,33^ which predicted that each chimeric peptide mimicked the conformation of the region within native IHF protein from which each was derived, i.e., beta sheets for the tip chimer peptide and alpha helices for the tail chimer peptide (Fig. 1d, f, respectively). Thus, there existed the potential to induce antibodies that recognized conformational, in addition to sequence-dependent, epitopes upon immunization with the chimeric peptides.

An important consideration in vaccine design is conservation of the target among bacterial strains or species. Specific to native IHF, all currently sequenced NTHi strains possess *ihfa* and *ihfb* alleles and compared with IHFA and IHFB as expressed by the prototype NTHi strain 86-028NP used in this work, there exists at least 93% amino acid identity (Supplementary Table 1). Moreover, the 20-mer regions within the DNA-binding tips of IHF that are incorporated into the tip chimer peptide showed 100% conservation among sequenced NTHi strains. As bacterial biofilms contribute to the chronicity of numerous diseases, we extended our evaluation to include *Burkholderia cenocepacia*, Uropathogenic *Escherichia coli, Klebsiella pneumoniae, Acinetobacter baumannii*, and *Pseudomonas aeruginosa*, high priority pathogens to public health*.* In silico analyses of the whole IHFA and IHFB proteins varied in the aforementioned bacteria from 40 to 63% compared with NTHi 86-028NP proteins, however, the tip epitopes were highly conserved and ranged from 80 to 95% identity (Supplementary Table 1). Therefore, the approach to focus on immunodominant and greatly conserved portion of IHF was predicted to have the potential to be broadly effective against biofilms formed by diverse strains of NTHi, and while beyond the scope of this current work, additional bacterial species of medical importance as well.

### Eradication of NTHi and resolution of mucosal biofilms from the middle ear

**Fig. 2:**
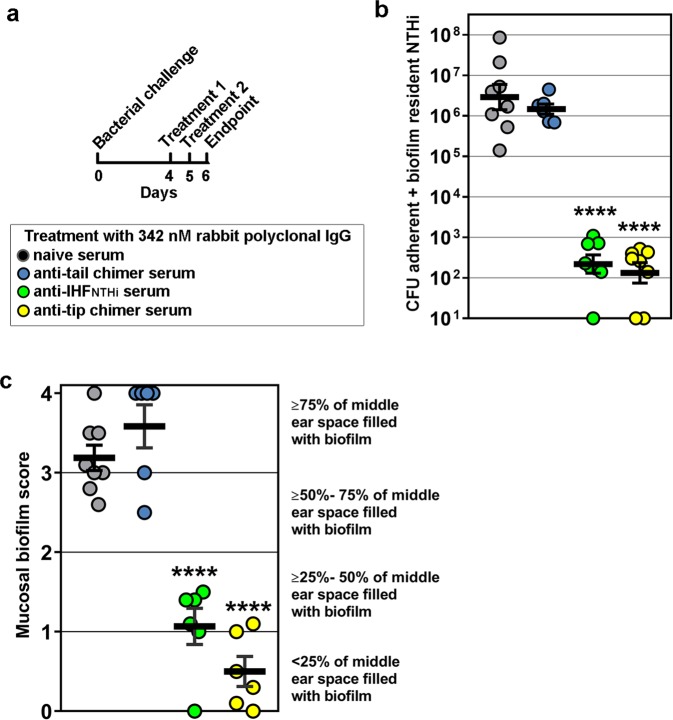
Quantitation of bacterial load and amount of mucosal biofilm in the middle ear 1 day after receipt of the second therapeutic dose of antibody. **a** Experimental protocol and symbols. **b** Eradication of NTHi adherent to the middle ear mucosa and within mucosal biofilms and (**c**) clearance of mucosal biofilms from the middle ear. Despite direct inoculation of both middle ears with NTHi to induce active otitis media and establish robust mucosal biofilms, a two-dose therapeutic regimen to deliver IgG against native IHF_NTHi_ or tip chimer peptide achieved a significant reduction in NTHi and clearance of mucosal biofilms within 1 day. *N* = 6–8 middle ears per cohort, values for individual middle ears and mean ± SEM are shown for each cohort. Limit of detection, 100 CFU NTHi. ****p ≤ 0.0001 compared with rabbit polyclonal IgG from naive serum or anti-tail chimer peptide serum. One-way ANOVA with Tukey’s multiple comparison tests

To assess the ability of antibody directed against the tip chimer to disrupt biofilms in the context of active disease, we utilized a chinchilla model of experimental otitis media wherein NTHi is inoculated directly into both middle ears where it forms a large biofilm within 4 days.^34,35^ Four days after bacterial challenge, rabbit polyclonal IgG was infused into each middle ear; this treatment was repeated 1 day later. Cohorts received either rabbit polyclonal IgG derived from native IHF_NTHi_ serum or anti-tip chimer serum with the same treatment administered to both ears of each animal. IgG from either anti-tail chimer serum or naive rabbit serum (NRS) was delivered to two separate negative control cohorts. A subset of animals within each of these four designated cohorts was killed 1 day after receipt of the second therapeutic antibody dose to determine the relative early effectiveness of treatment (Fig. 2a).

We first examined clearance of NTHi adherent to the middle ear mucosal epithelium and resident within mucosal biofilms. One day after receipt of the second therapeutic dose of rabbit polyclonal IgG, the middle ear mucosa along with any adherent mucosal biomass was collected in order to quantitate the number of adherent and biofilm-resident NTHi, respectively. Animals administered rabbit polyclonal IgG from anti-native IHF_NTHi_ serum or anti-tip chimer serum had a significant > 4.0-log reduction in NTHi (*p* ≤ 0.0001), compared with delivery of either anti-tail chimer or NRS (Fig. 2b). Thus, despite pre-establishment of a dense NTHi biofilm in the middle ear, antibody against either native IHF_NTHi_ (as we have shown previously^18^) or the tip chimer was highly effective to eradicate NTHi. There was, however, no significant difference between receipt of IgG from anti-native IHF_NTHi_ or anti-tip chimer sera.

We next assessed the resolution of established middle ear mucosal biofilms. The amount of residual middle ear mucosal biofilm was also estimated by blinded evaluators using an established scoring system to determine if antibody-mediated clearance of these communities was achieved. As expected based on culture data, 1 day after antibody therapy, NTHi mucosal biofilms filled ≥ 50% the middle ear space of animals treated with rabbit polyclonal IgG from either NRS or anti-tail chimer serum (Fig. 2c). In contrast, significantly less biofilm remained in cohorts administered IgG from either native IHF_NTHi_ or anti-tip chimer serum compared with either of the two control cohorts (*p* ≤ 0.0001). Moreover, in 83% (5/6) of middle ears infused with rabbit polyclonal IgG from anti-tip chimer serum, mucosal biofilms were largely cleared (as indicated by a score of ≤ 1.0), compared with only 33% (2/6) of middle ears in the cohort administered rabbit polyclonal IgG from anti-native IHF_NTHi_. While possibly biologically relevant, this latter difference was not, however, statistically significant. Collectively, rabbit polyclonal IgG derived from anti-native IHF_NTHi_ or anti-tip chimer serum induced an immediate significant reduction in amount of NTHi biofilm that remained within the middle ear, an outcome not achieved by receipt of rabbit polyclonal IgG from anti-tail chimer antiserum.

### Biofilm disruption was maintained without additional treatment administered

**Fig. 3:**
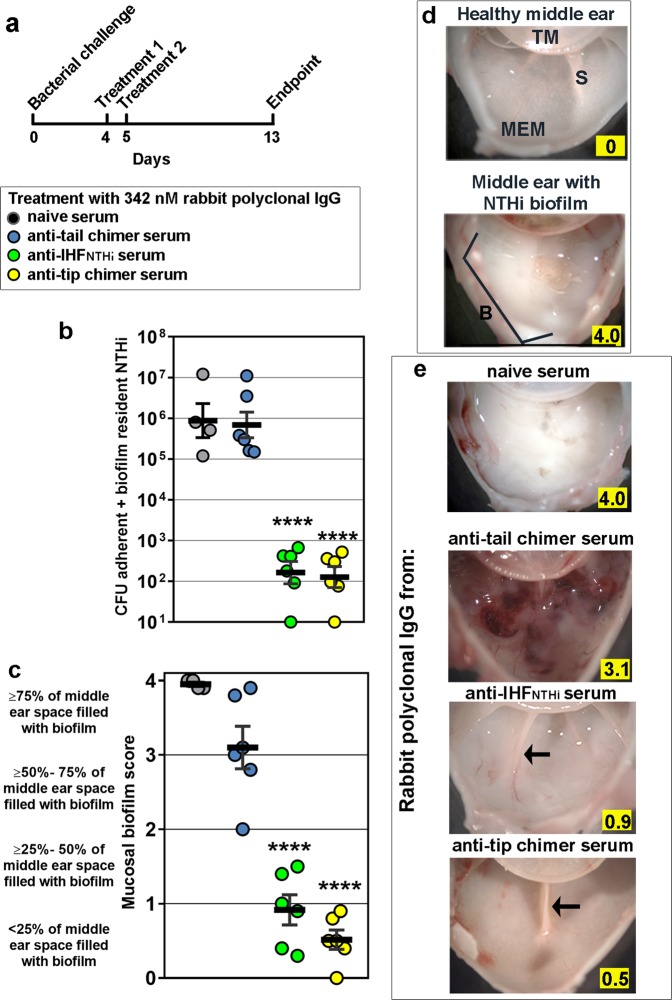
Quantitation of NTHi and qualitative assessment of amount of mucosal biofilm in the middle ear 8 days after receipt of the second therapeutic dose of rabbit polyclonal IgG. **a** Experimental protocol and symbols. **b** Eradication of NTHi and (**c**) clearance of mucosal biofilms from middle ears. **d** Example images of a healthy chinchilla middle ear and one with an NTHi mucosal biofilm, the mucosal biofilm score is highlighted in yellow within each image. TM tympanic membrane, S septae, MEM middle ear mucosa, B biofilm. **e** Visualization of mucosal biofilms, if present, within representative middle ears from each cohort; arrows indicate septae. Highlighted values within each image indicate mucosal biofilm score. Mucosal biofilms disrupted with rabbit polyclonal IgG from native IHF_NTHi_ serum or anti-tip chimer serum did not reform over an 8-day period after completion of antibody therapy, despite no additional treatments administered. *N* = 4–6 middle ears per cohort, values for individual middle ears and mean ± SEM are shown. Limit of detection, 100 CFU NTHi. ****p ≤ 0.0001 compared with rabbit polyclonal IgG from naive serum or anti-tail chimer serum. One-way ANOVA with Tukey’s multiple comparison tests

A second subset of animals from the same four cohorts, as described above, was evaluated 8 days later (Fig. 3a) to determine the relative durability of any achieved biofilm clearance in the absence of additional treatment.

As performed at the earlier time point, 8 days after completion of antibody therapy we quantitated the number of NTHi adherent to the middle ear mucosal epithelium and resident within mucosal biofilms. The adherent +biofilm-resident population of NTHi remained high within middle ears of animals administered rabbit polyclonal IgG from NRS (3.4 × 10^6^ CFU) or anti-tail chimer serum (2.6 × 10^6^ CFU) (Fig. 3b). Thus, treatment with IgG from anti-tail chimer serum did not provide a benefit, compared with the receipt of nonspecific NRS. Conversely, significantly fewer (< 300 CFU) adherent/biofilm-resident NTHi remained within the middle ears of animals that received rabbit polyclonal IgG enriched from anti-native IHF_NTHi_ or anti-tip chimer serum (*p* ≤ 0.0001 compared with anti-tail chimer or NRS). Thus, the residual number of NTHi within this niche detected 1 day after antibody therapy with rabbit polyclonal IgG from anti-native IHF_NTHi_ or anti-tip chimer serum (as shown in Fig. 2b) did not expand over an additional 8 days, despite no additional treatments administered (Fig. 3b).

As an additional assessment, we qualitatively evaluated the relative amount of mucosal biofilm within the middle ear. Eight days after completion of antibody therapy, significantly less mucosal biofilm remained in the middle ears of those animals that had received rabbit polyclonal IgG from anti-native IHF_NTHi_ or anti-tip chimer serum (*p* ≤ 0.0001), compared with receipt of IgG from anti-tail chimer serum or NRS (Fig. 3c). Between the two most effective therapeutic antibodies, a significantly greater proportion of middle ears that received rabbit polyclonal IgG from anti-tip chimer serum received a score of ≤ 1.0 (i.e., ≤ 25% of the middle ear space is occluded by a mucosal biofilm or no biofilm is present) compared with anti-native IHF_NTHi_ (*p* ≤ 0.05).

To provide visual validation of the biofilm scoring data presented in Fig. 3c, we compiled representative images of chinchilla middle ears 8 days after antibody therapy that depicted the mean value of the reported biofilm score for that cohort. As a baseline reference, an image of a healthy chinchilla middle ear with anatomical landmarks, such as the tympanic membrane, bony septae that extend out from under the tympanic annulus and middle ear mucosa, each annotated, represents a score of 0 (Fig. 3d, top panel). A middle ear with an established NTHi biofilm is also shown wherein the mucosal biofilm fills ≥ 75% of the normally sterile, air-filled space, thereby depicting a score of 4 (Fig. 3d, bottom panel). In this latter image, also note that visibility of the middle ear mucosa and septae are obscured by the presence of this dense NTHi biofilm.

Gross images of representative middle ears collected 8 days after antibody therapy with NRS or anti-tail chimer showed large, dense, white masses that represented NTHi mucosal biofilms (Fig. 3e, top two panels). Moreover, hemorrhagic foci and extensive mucosal inflammation were observed within the middle ears administered rabbit polyclonal IgG from anti-tail chimer serum (Fig. 3e, second panel). Whereas inoculation of NTHi into the middle ear does induce a host immune response due to the presence of a Gram-negative bacterium in the normally sterile middle ear space (see image of a middle ear with a biofilm and image after receipt of IgG from NRS), the extent of capillary dilation and hemorrhagic foci was greater after delivery of rabbit polyclonal IgG from anti-tail chimer serum, although the bacterial burden was comparable and the mean mucosal biofilm score was less than in middle ears administered IgG from naive serum. Thus, while the mechanism is not yet defined, delivery of the confirmed endotoxin-negative anti-tail chimer antibody did not resolve NTHi biofilms and resulted in an excessive inflammatory host immune response.

In contrast, mucosal biofilms were significantly reduced or completely eradicated in animals that received rabbit polyclonal IgG from anti-native IHF_NTHi_ or anti-tip chimer sera and thereby received a mean score ≤ 1.0 (Fig. 3e, bottom two panels). Normal anatomical landmarks (e.g., the bony septae that separate chambers within the tympanum, see arrows) were clearly visible within the middle ears of these latter animals, whereas they were obscured by mucosal biofilms in those that received IgG from NRS or anti-tail chimer serum. We also noted the ability to resolve the normal fine network of capillaries within the thin mucosa that lines the middle ear space in chinchillas treated with rabbit polyclonal IgG from anti-native IHF_NTHi_ or anti-tip chimer serum (Fig. 3e, bottom two panels).

### Relative kinetics of NTHi biofilm disruption as mediated by our panel of rabbit polyclonal IgG

**Fig. 4:**
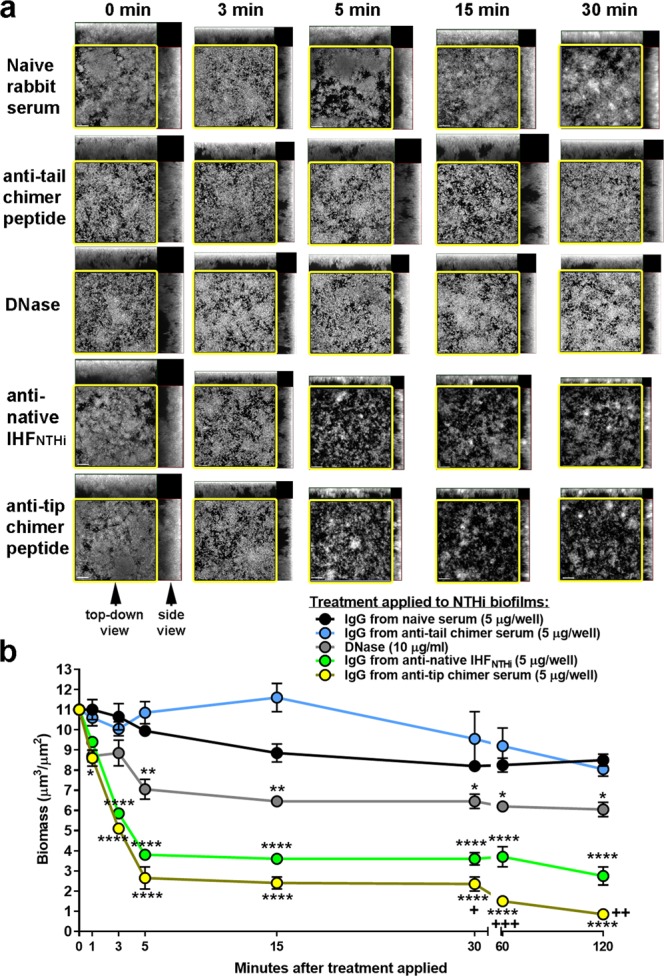
Rabbit polyclonal IgG derived from anti-tip chimer serum rapidly disrupted NTHi biofilms in vitro. **a** Representative images of NTHi biofilms incubated with rabbit polyclonal IgG derived from either naive or immune sera or DNase, captured by confocal scanning laser microscopy. Images were rendered as orthogonal projections to show a top–down view (to depict relative spacial distribution of the biofilm within the *x*–*y* planes and outlined in yellow box), as well as a side view (to depict relative biofilm height within the *z*-plane), as indicated by arrowheads; scale bars: 20 µm. **b** Reduction in biofilm biomass as determined by COMSTAT2 analysis. These data permitted discrimination between two highly effective biofilm disrupting treatments and revealed superiority of the tip chimer versus native IHF_NTHi_ protein to resolve NTHi biofilms in vitro. *N* = 3 assays, mean ± SEM are shown. Two-way ANOVA with Tukey’s multiple comparison tests. *p ≤ 0.05 versus IgG from naive serum and anti-tail chimer serum; **p ≤ 0.01 versus IgG from naive serum and anti-tail chimer serum; ****p ≤ 0.0001 versus IgG from naive serum and anti-tail chimer serum. ^+^*p* ≤ 0.05 versus IgG derived from anti-IHF_NTHi_; ^+ +^*p* ≤ 0.01 versus IgG from anti-IHF_NTHi_, ^+ + +^*p* ≤ 0.001 versus IgG from anti-IHF_NTHi_

To overcome the inability to resolve differences in therapeutic efficacy between cohorts administered rabbit polyclonal IgG from anti-native IHF_NTHI_ serum or anti-tip chimer serum, we now endeavored to assay the relative ability of these two antisera to disrupt an established 24-hr NTHi biofilm over time, via use of a chamber slide assay in vitro. Representative images revealed that incubation with either rabbit polyclonal IgG from naive serum or anti-tail chimer serum had little if any disruptive effect on an NTHI biofilm (Fig. 4a, top 2 rows, respectively). Treatment with DNase (Fig. 4a, 3rd row) resulted in limited biofilm disruption. Notably, however, markedly less NTHi biofilm remained after incubation with either rabbit polyclonal IgG from anti-native IHF_NTHi_ serum or anti-tip chimer serum, an outcome that was evident within 3 min (Fig. 4a, bottom 2 rows) and maintained through the 120-min assay period.

Quantitation of the relative amount of biofilm biomass that remained after treatment is shown in Fig. 4b. Regardless of incubation time, treatment with rabbit polyclonal IgG from NRS or anti-tail chimer serum had little disruptive effect. DNase, used herein to enzymatically break down the eDNA matrix known to be an extensive component of the biofilm extracellular polymeric substance, resulted in some biofilm disruption that was significantly greater than that induced by either rabbit polyclonal IgG from naive serum or anti-tail chimer sera (*p* ≤ 0.05 or *p* ≤ 0.01). However, significantly greater biofilm collapse was achieved by incubation with rabbit polyclonal IgG from anti-IHF_NTHi_ serum or anti-tip chimer serum that was observed for this latter serum by the 1 min time point (*p* ≤ 0.05). Notably, 3 min after application of rabbit polyclonal IgG derived from anti-native IHF_NTHi_ serum or anti-tip chimer serum to the biofilms, significant disruption was observed (*p* ≤ 0.0001 compared with rabbit polyclonal IgG from NRS or anti-tail chimer serum). This disruption was rapid and sustained for the duration of the assay period.

Thereby, unlike the chinchilla model in which the outcome measures had limited ability to discriminate between the two most effective treatments, this in vitro kinetic assay allowed us to now discriminate subtle differences in biofilm disruption ability of IgG antibody from anti-native IHF_NTHi_ serum versus anti-tip chimer serum. Whereas both of these treatments were highly effective, rabbit polyclonal IgG from anti-tip chimer serum showed biofilm disruptive efficacy sooner, and was significantly more disruptive than anti-native IHF_NTHI_ within 30 min of exposure (*p* ≤ 0.05). This significant difference was maintained throughout the remainder of the assay (e.g., 60 and 120 min) (Fig. 4b, c).

### Assessment of the natural immune response to the DNABII protein IHF

As detailed above, as well as in earlier studies,^14,16–19^ we hypothesized that the natural adaptive immune response to the DNABII proteins within any pathogenic biofilm was likely directed toward non-protective epitopes, specifically those in the exposed carboxyl-terminal tail region within the α-subunit and β-subunit due to masking of immunoprotective domains by intercalation with eDNA. If correct, we reasoned that this might provide an explanation as to why humans did not develop an effective adaptive immune response that would enable them to clear these recalcitrant biofilms. If true, this would mean that sera recovered from human subjects would naturally show a skewed response toward the non-protective tail domains of the DNABII proteins.

**Fig. 5:**
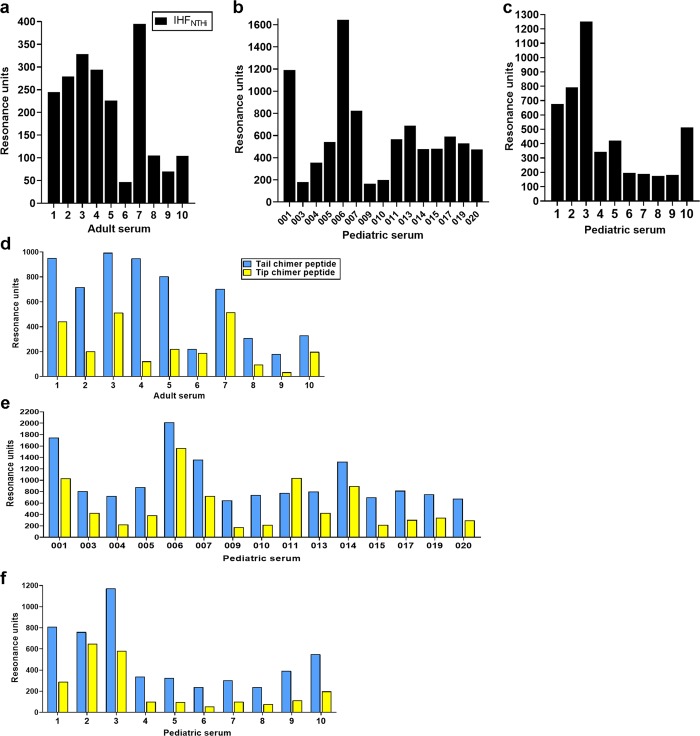
The natural adaptive immune response in adults and children is predominantly directed against the non-protective tail region of IHF. Reactivity of antibodies in serum from (**a**, **d**) healthy adults and (**b**, **c**, **e**, **f**) children with chronic otitis media to (**a**–**c**) native IHF_NTHi_ or (**d**–**f**) tip and tail chimer peptides as assessed by surface plasmon reference (SPR) and reported as resonance units (RU). Baseline-corrected values are shown for each specimen assayed versus individual flow cells to which IHF_NTHi_, tip chimer or tail chimer were immobilized. The natural human immune response is predominantly shifted toward the non-protective tail region of IHF

To test this premise, we examined the preferential binding of antibodies in serum from healthy adults (who likely had experienced diseases with a biofilm component throughout their lifetime) and children with active chronic otitis media (and thereby with an active biofilm disease at the time of serum collection) to native IHF_NTHi_ and either the tail chimer or tip chimer by surface plasmon resonance (SPR). First, we determined whether antibodies directed against IHF were present within these panels of sera. Each of the ten adult sera demonstrated recognition of native IHF (Fig. 5a). Moreover, antibodies in each of the 25 pediatric serum samples from children undergoing tympanostomy tube insertion for chronic otitis media with effusion wherein antibiotic therapy had repeatedly failed also recognized native IHF_NTHi_ (Fig. 5b, c). One set of 15 frozen sera was retrieved from an archived collection that represented a span of 3 years in the period from 2007 to 2010 (Fig. 5b, e), whereas a second set of ten fresh sera was assayed immediately after collection in the operating room (recovered in the years 2017–2018) (Fig. 5c, f). Of note, antibodies from children with otitis media had significantly greater reactivity to IHF_NTHi_ compared with the adult sera (mean values: 546 vs. 209 resonance units, respectively; *p* ≤ 0.01), likely a consequence of an active disease process in the former population.

With reactivity to native IHF demonstrated, we next discerned whether these antibodies would further show preferential recognition to either the tail region of IHF or the DNA-binding tip region of the protein. Antibodies in each of the ten sera from healthy adults did indeed demonstrate preferential recognition of the tail chimer, and the magnitude of this recognition was significantly greater compared with recognition of the tip chimer (mean values: 616 resonance units to tail chimer vs. 253 resonance units to tip chimer; *p* ≤ 0.01) (Fig. 5d). When we evaluated previously frozen pediatric sera, 14 of the 15 samples tested (93%) showed significantly greater recognition of the tail chimer peptide over recognition of the tip chimer peptide (mean values: 985 RU vs. 551 RU, respectively; *p* ≤ 0.01) (Fig. 5e). Similarly, antibodies within 100% (10/10) freshly collected pediatric sera showed significantly greater recognition of the IHF tail chimer compared with the tip chimer (mean values: 513 resonance units *vs*. 226 resonance units, respectively; *p* ≤ 0.05) (Fig. 5f).

As an additional assessment, an ELISA was performed to determine the 50% binding point (B50%) for each serum after incubation of serial dilutions of serum with IHF protein, tip or tail chimer peptides. Whereas reactivity in the SPR assay takes into account antibody affinity and avidity under continuous flow, the ELISA could permit detection of weaker antibody binding by virtue of the static experimental conditions. As observed by SPR, antibody in all 10 healthy adults and all 25 children with active otitis media recognized native IHF, although by ELISA, the relative binding was comparable between the two groups (B50%: 22 for adult sera vs. 23 for the pediatric sera). Nonetheless, and as demonstrated by SPR, there was significantly greater recognition of the tail chimer peptide compared with the tip chimer by both the adult sera (B50%: 30 versus 19, respectively; *p* ≤ 0.05) and pediatric sera (B50%: 37 vs. 21 respectively; *p* ≤ 0.05).

Collectively, antibodies in serum from 100% healthy adults and from 96% (24/25) of children with chronic otitis media demonstrated significantly greater recognition of the tail chimer, compared with the tip chimer. Thus, the natural adaptive human immune response appeared to indeed be predominantly directed toward non-protective epitopes within the tail region of this DNABII protein. This observation contributed to our original concern that whereas immunization with a native DNABII protein was effective in an animal model, use of the native protein in a human subject was potentially problematic, as there would be strong potential to induce an anamnestic response to the non-protective domains. Whereas use of a native DNABII protein would also induce a response to the tip regions, the overall response would not be strongly (and perhaps more exclusively) directed toward the immunoprotective tip domains. Thereby, we reasoned that use of the tip chimer would have a greater likelihood of success in redirecting the immune response as desired, and thereby avoid this potential confounding issue with regard to use of the native protein.

### Redirection of the natural immune response to immunoprotective domains of IHF in a mammalian host

**Fig. 6:**
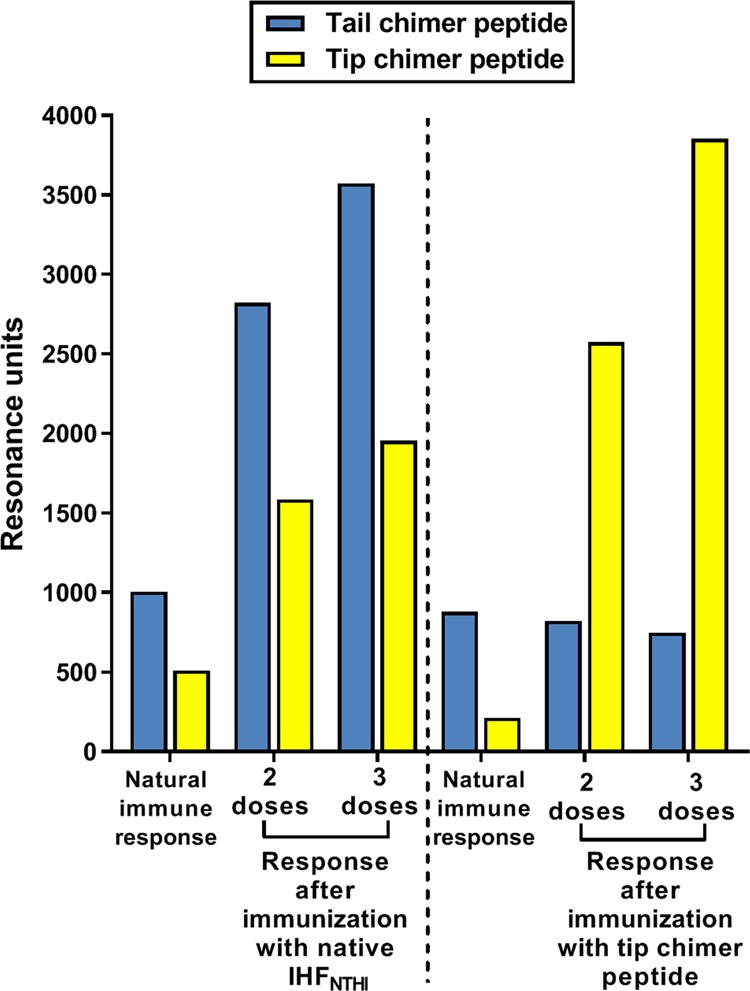
Redirection of the natural mammalian immune response to protective domains of IHF. Reactivity of antibodies in serum from chinchillas immunized with native IHF_NTHi_ or tip chimer peptide to tip or tail chimer peptides as determined by surface plasmon reference (SPR). Baseline-corrected values are shown. Immunization with the tip chimer was highly effective to re-focus the natural immune response to IHF away from non-protective domains, and exclusively toward immunoprotective domains, as by design

To examine this premise, we first ascertained whether chinchillas, which experience their own diseases with a pathogenic biofilm component,^36^ also demonstrate a naturally skewed adaptive immune response to the non-protective tail portions of DNABII proteins. As shown in Fig. 6 for two outbred (nonspecific pathogen free) chinchillas, they too demonstrated a skewed preferential recognition of the tail chimer, similar to humans. Upon active immunization with native IHF_NTHi_, there was an increased recognition of both the tip and the tail chimers as expected, however, recognition of the tail chimer was 1.8-fold greater after each of two doses delivered compared with the tip chimer. Conversely, upon active immunization with the tip chimer immunogen, there was 3.1-fold to 5.1-fold increase in preferential recognition of the tip chimer, whereas there was no measurable increase in recognition of the tail chimer. These data provided strong support to our rationale for use of the tip chimer over native DNABII protein as a means to strongly re-direct the natural adaptive immune response toward the immunoprotective tip domains of a DNABII protein and away from the non-protective tail domains.

## Discussion

Biofilm infections are responsible for a significant global burden of disease with considerable socioeconomic impact, as well as diminished quality of life. These microbial communities are the preferred lifestyle for bacteria and 80% of human infections involve a biofilm component,^1,37^ including those within the upper and lower respiratory tracts,^38–41^ oral cavity,^42,43^ gastrointestinal tract^44,45^, and urogenital tract.^46,47^ Biofilm-resident bacteria are ~1000-fold more resistant to the action of antibiotics than their planktonic counterparts^48^ due to a combination of reduced growth rate and protection from exposure afforded by the EPS. Therefore, it is necessary to develop novel and powerful ways to both eradicate existing biofilms or where possible, prevent their formation. This could be achieved with either an effective therapeutic approach, a preventative vaccine approach, or a therapeutic vaccine approach. To be the most broadly effective, any such strategy would ideally need to target a universal biofilm component, expressed by most, if not all predominant bacterial pathogens. We believe we have identified a potential candidate that meets these criteria, the DNABII family of DNA-binding proteins.

Based on prior experiences in design and refinement of highly immunogenic and protective protein and peptide immunogens,^49,50^ we reasoned that domains of the DNABII proteins are obscured when bound to eDNA within the biofilm matrix and thereby, may not be available for immune recognition. This hypothesis was confirmed in a prior study, wherein chinchillas were immunized with either purified native IHF or IHF that was pre-complexed to DNA (to represent the form of DNABII proteins that exists in a biofilm). NTHi biofilms persisted in the middle ears of animals immunized with IHF pre-complexed to DNA, whereas immunization with purified native IHF eradicated the established biofilms.^14^

Epitope-mapping studies using serum collected from chinchillas immunized with IHF pre-complexed to DNA (collected prior to bacterial challenge) subsequently showed that whereas these antibodies were primarily directed against the accessible “tail” region of IHF, antibodies against native IHF protein were instead focused on the DNA-binding “tip” regions.^16^ Thus, the tip regions within the α-subunit and β-subunit of native IHF contained immunoprotective domains. To exploit this fact, murine monoclonal antibodies directed exclusively against the DNA-binding tip regions within either the α-subunit or β-subunit of IHF were generated and found to disrupt established biofilms in vitro and in vivo, whereas monoclonal antibodies against the tail region (either α-subunit directed or β-subunit directed) did not.^18^ Antisera directed toward the α-tip and the β-tip were equally effective to disrupt the established biofilms, and when tested as a cocktail of monoclonal antibodies, biofilm disruption was even greater.

Thus, the approach to target the DNA-binding tip domains of IHF_NTHi_ with rabbit polyclonal IgG was highly effective to reduce both quantity of NTHi and amount of mucosal biofilm that remained within the middle ear, an outcome observed immediately and maintained without additional treatment. Collectively, the data obtained via use of the chinchilla model of experimental otitis media supported our rationale for the design of the tip chimer as a highly effective immunogen, but these data did not yet provide conclusive proof of superiority of use of the tip chimer over native IHF protein as an immunogen.

Since these data supported a hypothesis wherein during natural disease, the adaptive immune response was directed toward the exposed tail regions of native IHF and thus was ineffective, we came to two conclusions: (1) humans likely innately possessed an existing tail-biased response and (2) immunization with the native protein would thereby continue to drive that ineffective response. To expand upon this line of investigation, we first tested serum from humans to see if the former hypothesis was true. To that end, three panels of sera were examined: two panels from children with active, chronic otitis media at the time of collection and one from heathy adults, who presumably had a biofilm-related disease, if not several, at some point in their lives. In sum, antibodies in 34 of 35 human sera tested showed preferential recognition of the tail chimer. Only one child showed preferential recognition of the tip chimer. Collectively, these data provided strong support for the design of a chimeric tip peptide immunogen to capitalize on the fact that use of a cocktail of monoclonal antibodies directed against immunoprotective domains within the α-subunit and β-subunit of IHF was even more effective than use of either monoclonal alone.^18^ This additive effectiveness is due, in part, to the fact that whereas highly similar, these two regions are not identical in sequence.

As proof of principle, herein we demonstrated that antibodies against the tip chimer were highly effective to promote NTHi clearance and resolve established biofilms from the middle ears of chinchillas in the context of experimental otitis media. Specific to early versus late outcomes for each cohort, between 1 and 8 days after completion of antibody therapy, whereas a minor decrease in number of NTHI was observed in the middle ears of animals administered IgG from naive rabbit serum, these bacterial counts remained > 10^6^ total CFU NTHi present. Moreover, mucosal biofilms established in the middle ear prior to antibody therapy endured, without clearance by the host, and > 75% of the middle ear space remained filled with biomass for the duration of the study. Therefore, given that in this model system relative bacterial concentration and mucosal biofilms persisted in animals treated with nonspecific naive rabbit serum, any improvement or worsening in disease outcomes in the remaining three cohorts would be attributable to the effect of antisera that target IHF and specifically, the “tail” or “tip” domains within the native protein.

Receipt of IgG against the tail chimer did not promote clearance of NTHi from the middle ear, as > 10^6^ CFU NTHi remained present, comparable in quantity to the cohort administered IgG from naive rabbit serum. Whereas a slight decrease in mucosal biomass score was observed 8 days after antibody therapy compared with day 1 (3.1 vs. 3.6, respectively), the reduction in mean value was largely driven by one of the six middle ears evaluated at this time point, yet bacterial counts for this particular middle ear persisted at > 10^5^ CFU which was comparable with the mean for the entire cohort. Thus, whereas IgG against the tail chimer, which targets non-protective domains within native IHF_NTHi_, did not facilitate clearance of NTHi or resolution of middle ear mucosal biofilms, IgG against native IHF_NTHi_ provided an immediate benefit, shown by a significant reduction in quantity of NTHi present in the middle ear and clearance of mucosal biofilms 1 day after completion of antibody therapy, compared with negative controls. While not statistically significant, an additional two-fold reduction in number of NTHi was also observed after further 8 days and mean bacterial counts approached the limit of detection. Mucosal biofilms similarly continued to decrease over time. Therefore, NTHi did not re-establish biofilms within the middle ear after delivery of two doses of IgG against native IHF_NTHi_, despite no additional treatment.

Delivery of IgG from anti-tip chimer serum also provided a significant benefit, compared with either negative control cohort. Nominal bacterial counts were detected at each time point, the values of which were at the limit of detection. Mean mucosal biofilm scores were 0.5 (i.e., < 25% of the middle ear space was occluded by biofilm) at each time point. Thus, the approach to target the DNA-binding tip domains of IHF_NTHi_ with rabbit polyclonal IgG was highly effective to reduce both quantity of NTHi and amount of mucosal biofilm that remained within the middle ear, an outcome observed immediately and maintained without additional treatment.

While very promising, the chinchilla model did not yet facilitate our ability to discriminate the relative superiority of the tip chimer versus native IHF as either a potential therapeutic treatment or as an immunogen. To begin to provide this discrimination, we utilized a sensitive in vitro kinetic biofilm disruption assay. By this technique, a clear and significant superiority of the polyclonal anti-tip chimer serum was demonstrated. Therefore, our rationale for the design of the tip chimer as a means to redirect the natural immune response to a native DNABII protein was validated. Tip chimer antibodies effectively eradicated biofilms within the middle ears of chinchillas after two doses, with only a single concentration tested to date. Importantly, and under full consideration, as we continue to develop this DNABII-targeted approach, is careful crafting of the active immunization strategy to both capitalize on its power yet obviate the potential for collateral damage by disruption of “good” biofilms (i.e., curing a wound infection but causing diarrhea for example). This can be achieved by local delivery of either therapeutic antibodies or immunogen (e.g., direct delivery to a specific anatomical niche/wound site or transcutaneous immunization for example), titration of dosing and careful adjuvant selection and should be defined by disease, access to site of disease, microbes involved, immune status, and age of the host, among other considerations.

Combatting biofilm diseases will require new approaches, solidly based on a firm understanding of biofilm biology. This should include approaches to eradicate biofilms as well as prevent their formation either directly or via a combinatorial approach. These approaches cooperatively have potential to tip the balance in favor of the host.

## Methods

### Synthetic peptides and polyclonal rabbit antisera

Amino acid sequences that represented the individual DNA-binding tip regions of IHF_NTHi_ for the α-subunit and β-subunit were bridged with a flexible GPSL turn linker peptide to permit each epitope to fold independently.^29,49^ Amino acid sequences that represented the tail regions within the α-subunit and β-subunit of IHF_NTHi_ were similarly linked. In order to optimally orient immunodominant regions, the amino acid sequences for each tip region within the α-subunit and β-subunit and the tail region within the α-subunit were oriented N-to-C terminus, whereas the sequence for the β-subunit tail region was aligned C-to-N terminus.^16^ Synthesis, purification, and sequence confirmation were performed by Ohio Peptide, LLC (for tip chimer), and Genscript (for tail chimer), and both chimeric peptides were confirmed to be ≥ 95% pure. Generation of polyclonal antisera against native IHF_NTHi_ protein, tip chimer, and tail chimer was performed at Rockland Immunochemical, Inc. Briefly, white New Zealand rabbits were injected subcutaneously with 250 µg protein or peptide admixed with complete or Incomplete Freund’s adjuvant; three doses were delivered at 21-day intervals. Ten days after receipt of the final dose, blood was collected and processed to separate serum. Rabbit polyclonal IgG was then enriched as the primary antibody isotype that potentiates adaptive immune responses by passage of whole serum through rProtein A Protein G GraviTrap columns according to the manufacturer’s instructions (GE Healthcare Life Sciences). Bacterial endotoxin test via Limulus Amebocyte lysate test was performed on all antibody lots prior to use.

### Bacterial strain and biofilm disruption assays in vitro

NTHi strain 86-028NP is a minimally passaged clinical isolate cultured from the nasopharynx of a child with chronic otitis media. NTHi biofilms were established in brain heart infusion broth supplemented with 2 μg each of β-nicotinamide adenine dinucleotide (β-NAD) and heme per ml for 24 hr in eight-well chambered cover glass slides as described.^51^ To examine the kinetics of antibody-mediated biofilm collapse, biofilms were incubated with 5 μg rabbit polyclonal IgG derived from anti-native IHF_NTHi_ antiserum, anti-tip chimer serum, naive rabbit serum or 10 μg DNase per well. Wells in which treatment was applied, and then immediately removed, served as data for time point zero. Biofilms were stained with FM 1-43FX membrane stain according to the manufacturer’s instructions (Molecular Probes). All biofilms were then fixed with 16% paraformaldehyde, 2.5% glutaraldehyde, and 4% acetic acid in 0.1 M phosphate buffer (pH 7.4) for 24 h at 25 °C prior to visualization on a Zeiss 510 or 800 Meta laser scanning confocal microscope.^18^ Images were rendered with Zeiss Zen 2012 software, and biofilm biomass calculated with COMSTAT2 software.^52^ Experiments were performed a minimum of three times on separate days, and the mean ± SEM for bacterial biomass was reported.

### Biofilm disruption in vivo

Thirty-two chinchillas (*Chinchilla lanigera*; Rauscher’s Chinchilla Ranch, LLC, LaRue, Ohio) were enrolled and randomly divided into four cohorts of six to eight animals, each based on body weight. Both male and female animals were enrolled. Experimental otitis media was first induced in all animals by direct inoculation of both middle ears with 1000 CFU NTHi strain 86-028NP diluted in sterile, pyrogen-free 0.9% saline. Four days later, when classic clinical signs of OM are evident (as observed by video otoscopy and tympanometry) and NTHi has established a mucosal biofilm that fills ≥ 50% of the middle ear space in > 88% of middle ears in this model,^35^ 342 nM of rabbit polyclonal IgG derived from anti-native IHF_NTHi_ serum or anti-tip chimer serum was instilled into each chinchilla middle ear in a volume of 100 μl; both middle ears from the same animal received the identical antibody treatment.^18^ This specific concentration was selected to be equivalent to that used in biofilm disruption assays in vitro and represents the concentration of IgG-specific anti-IHF within polyclonal serum as published earlier.^16^ Cohorts administered 342 nM rabbit polyclonal IgG from tail chimer serum or naive rabbit serum served as negative controls. A second treatment was delivered 24 h later. One and 8 days after receipt of the second dose, three to four animals per cohort were humanely euthanized by barbiturate overdose. Chinchilla work was performed in accordance with federal, state, and institutional guidelines, and under protocol 01304AR approved by the Nationwide Children’s Hospital Institutional Animal Care and Use Committee.

Each bulla was dissected, and the middle ear washed with 1.0 ml 10 mM phosphate buffered saline (pH 7.4; PBS) to remove nonadherent NTHi within this niche. Images of adherent mucosal biofilm, if present, were captured with a Zeiss SV6 dissecting microscope and rendered with Zeiss Zen software. The middle ear mucosa and adherent NTHi mucosal biofilm within each middle ear were then collected and weighed. A volume of 0.5 ml sterile 10 mM phosphate buffered saline was then added and each specimen individually homogenized with an Omni tissue homogenizer and sterile 7 × 155 mm stainless-steel generator probes, using a setting of 18,000 rpm for 30 s. This procedure results in thorough disruption of the tissue and biofilm based on visual inspection. Homogenized mucosae were then serially diluted and plated on to chocolate agar to quantitate the number of adherent NTHi. In addition, to evaluate the relative quantity of mucosal biofilm that remained within the middle ear of each animal, images were randomized and scored by eight blinded reviewers not involved in the study using an established rubric whereby 0 = no mucosal biofilm visible, 1 = < 25% of middle ear space occluded by mucosal biofilm, 2 = ≥ 25–50% occluded, 3 = ≥ 50–75% occluded, 4 = ≥ 75–100% occluded.^18,35^

### Immunization of chinchillas with native IHF_NTHi_ or tip chimer peptide

To generate immune serum in chinchilla for assessment of immunoreactivity by surface plasmon resonance, two adult chinchillas were enrolled. Formulations consisted of 10 μg native IHF_NTHi_ protein or 10 μg tip chimer peptide. Protein/peptide was admixed with the adjuvant LT(R192/L211A), a double mutant of *E. coli* heat-labile enterotoxin (or “dmLT”; a generous gift from Dr. John D. Clements at Tulane University). Two amino acid substitutions within the alpha subunit (at amino acids 192 and 211) render the molecule nontoxic, compared with the native holotoxin; however, immunostimulatory properties are retained.^53^ Animals were immunized by subcutaneous injection delivered along the rear flanks. Ten days after the first dose was administered, the second immunization was performed. Chinchilla work was performed in accordance with federal, state, and institutional guidelines, and under protocol #01304AR, approved by the Nationwide Children’s Hospital Institutional Animal Care and Use Committee.

### Surface plasmon resonance

The natural immune response of healthy adults, children with chronic otitis media, naive chinchillas, and chinchillas that had been immunized with native IHF_NTHi_ or the tip chimer was discerned via surface plasmon resonance (SPR) using a Biacore 3000 instrument (GE Healthcare). A CM5 reagent grade sensor chip (GE Healthcare) was used to immobilize the tip chimer or the tail chimer peptides to individual flow cells via amine coupling chemistry. Once the peptide was bound, 15 µl of each serum was injected neat across the sensor chip surface at a flow rate 5 μl/min, a sample volume and flow rate that achieves equilibrium. The surface was regenerated with 5 µl of NaOH 50 buffer (GE Healthcare). HBS-EP buffer (GE Healthcare) served as the running buffer. Relative binding of serum antibodies was determined by comparison of resonance unit (RU) values before and after each antibody injection cycle. One panel of pediatric serum from children with chronic otitis media was retrieved from an archived specimen collection (collected from 2007 to 2010). To control for potential loss of antibody reactivity due to long-term storage of these specimens, a second panel of fresh pediatric sera from children with chronic otitis media was obtained (collected from 2017 to 2018) and assayed immediately after blood draw (not frozen). Serum from healthy adults was collected from 2017 to 2018. Adult blood was retrieved from the blood donor program at Nationwide Children’s Hospital. Collection of pediatric blood was performed after receipt of written Informed Consent and under protocols approved by the Nationwide Children’s Hospital Institutional Review Board (protocols #0312HS196, #IRB17-00968).

### Enzyme-linked immunosorbent assay (ELISA)

An ELISA was performed to determine the B50% of antibodies in the adult and pediatric sera to native IHF protein and chimeric peptides in a static assay format. Native IHF protein, tip chimer, or tail chimer peptides were adsorbed at 0.2 µg per well on to Nunc C-bottom MaxiSorp 96-well plates for 16 h at 4 °C. Wells were then blocked with 0.5% bovine serum albumin in 10 mM phosphate buffered saline (PBS), pH 7.4 for 1 h at 37 °C. After three washes of 200 µl 0.05% Tween 20 in 10 mM PBS, twofold serial dilutions of each serum specimen (1:2 to 1:64) were added and incubated for 16 h at 4 °C. Wells were washed as before and incubated with goat anti-human IgG Fc fragment (Bethyl Laboratories, #A80-304P) diluted 1:1000 in 10 mM PBS for 1 h at 37 °C. Wells were washed three times, and color developed with Thermo Scientific 1-Step Ultra TMB and the reaction stopped with 2 M H_2_SO_4_. Absorbance at 450 nM was determined on a BMG Labtech FLUOstar Omega plate reader. The data were entered into GraphPad Prism, normalized and analyzed via nonlinear regression to determine the B50% for each serum.

### Statistics

Statistically significant differences were calculated using GraphPad Prism 8 (GraphPad Software, Inc.). Differences in NTHi biofilm biomass after incubation with polyclonal rabbit sera in vitro were determined by one-way ANOVA and Tukey’s multiple comparisons test. Differences in CFU NTHi adherent to the middle ear mucosa and within mucosal biofilms were calculated by Mann–Whitney test. Resolution of mucosal biofilms in vivo was analyzed by one-way ANOVA and Tukey’s multiple comparisons test. To determine differences in proportion of middle ears that scored ≤ 1.0 for residual amount of mucosal biofilm, Mantel–Cox log-rank test was performed. For all analyses, a *p-*value ≤ 0.05 was considered significant.

### Reporting summary

Further information on experimental design is available in the Nature Research Reporting Summary linked to this article.

## Supplementary information

Reporting summary

Supplementary Table 1

## Data Availability

The authors declare that the main data supporting the findings of this study are available within the article and its Supplementary Information files.
