# Supplementary material for: Redirecting the immune response towards immunoprotective domains of a DNABII protein resolves experimental otitis media
Source: NPJ Vaccines. 2019 Oct 14;4:43. doi: 10.1038/s41541-019-0137-1 (PMC6791836; doi:10.1038/s41541-019-0137-1)
Supplement: Supplementary file 2 — Supplementary Table 1 [file 41541_2019_137_MOESM2_ESM.docx]

| **Percent amino acid identity compared to NTHi 86-028NP** | | | | | | |
| --- | --- | --- | --- | --- | --- | --- |
|  | **IHF subunit** | | **20-mer incorporated into the tip chimer peptide** | | **20-mer incorporated into the tail chimer peptide** | |
| **Strain** | **α- subunit** | **β-subunit** | **α-tip** | **β-tip** | **α-tail** | **β-tail** |
| NTHi 86-028NP | 100.0 | 100.0 | 100.0 | 100.0 | 100.0 | 100.0 |
| NTHi R2846 | 100.0 | 100.0 | 100.0 | 100.0 | 100.0 | 100.0 |
| NTHi R3021 | 100.0 | 97.8 | 100.0 | 100.0 | 100.0 | 95.0 |
| NTHi 22.4-21 | 100.0 | 100.0 | 100.0 | 100.0 | 100.0 | 95.0 |
| NTHi PittAA | 98.9 | 100.0 | 100.0 | 100.0 | 100.0 | 95.0 |
| NTHi PittEE | 98.9 | 100.0 | 100.0 | 100.0 | 100.0 | 100.0 |
| NTHi PittHH | 93.7 | 100.0 | 100.0 | 100.0 | 90.0 | 95.0 |
| *H.* *influenzae* Rd KW20 | 98.9 | 100.0 | 100.0 | 100.0 | 100.0 | 90.0 |
| *B.* *cenocepacia* | 57.2 | 47.8 | 85.0 | 80.0 | 70.0 | 35.0 |
| *E.* *coli* UTI89 | 63.1 | 56.4 | 95.0 | 80.0 | 85.0 | 30.0 |
| *K.* *pneumoniae* | 63.1 | 56.4 | 95.0 | 80.0 | 85.0 | 30.0 |
| *A. baumannii* | 53.5 | 48.4 | 90.0 | 85.0 | 55.0 | 15.0 |
| *P.* *aeruginosa* | 40.0 | 59.8 | 85.0 | 90.0 | 65.0 | 65.0 |

Supplementary Table 1. Percent amino acid identity of IHF subunits and 20-mer epitopes incorporated into tip chimer and tail chimer peptide immunogens
